# Edible Bird’s Nest Ameliorates Dextran Sulfate Sodium-Induced Ulcerative Colitis in C57BL/6J Mice by Restoring the Th17/Treg Cell Balance

**DOI:** 10.3389/fphar.2021.632602

**Published:** 2021-04-21

**Authors:** Yaohua Fan, Yanqun Fan, Kunfeng Liu, Piyanuch Lonan, Feng Liao, Yuhang Huo, Xiaohua Zhong, Yueliang Liang, Yaxin Wang, Shaozhen Hou, Xiaoping Lai, Geng Li, Weihong Kuang

**Affiliations:** ^1^ Traditional Chinese Medicine Innovation Research Center, Shenzhen Hospital of Integrated Traditional Chinese and Western Medicine, Guangzhou University of Chinese Medicine, Shenzhen, China; ^2^ Xiamen Yan Palace Seelong Food Co., Ltd., Xiamen, China; ^3^ Laboratory Animal Center, Guangzhou University of Chinese Medicine, Guangzhou, China; ^4^ Guangzhou Tongkang Pharmaceutical Co., Ltd., Guangzhou, China; ^5^ School of Pharmaceutical Sciences, Guangzhou University of Chinese Medicine, Guangzhou, China; ^6^ Guangdong Provincial Key Laboratory of New Drug Development and Research of Chinese Medicine, Mathematical Engineering Academy of Chinese Medicine, Guangzhou University of Chinese Medicine, Guangzhou, China; ^7^ Guangdong Key Laboratory for Research and Development of Natural Drugs, Key Laboratory of Research and Development of New Medical Materials of Guangdong Medical University, School of Pharmacy, Guangdong Medical University, Dongguan, China

**Keywords:** ulcerative colitis, edible bird’s nest, immune regulation, Th17 cells, treg cells

## Abstract

Ulcerative colitis (UC) is a type of inflammatory bowel disease (IBD) with a complex aetiology that commonly recurs. Most drugs for UC treatment interfere with metabolism and immune responses, often causing some serious adverse reactions. Therefore, the development of alternative treatments, including nutritional supplements and probiotics, have been one of the main areas of current research due to fewer side effect. As both a Chinese medicine and a food, edible bird’s nest (EBN) has high nutritional value. Modern pharmacological studies have shown that it has anti-inflammatory, immunoregulatory, antiviral and neuroprotective effects. In this study, UC was induced with dextran sulfate sodium (DSS) to investigate the protective effect of EBN on colitis mice and the related mechanism. The body weight, faecal morphology and faecal occult blood results of mice were recorded every day from the beginning of the modelling period. After the end of the experiment, the length of the colon was measured, and the colon was collected for histopathological detection, inflammatory factor detection and immunohistochemical detection. Mouse spleens were dissected for flow cytometry. The results showed that in mice with colitis, EBN improved symptoms of colitis, reduced colonic injury, and inhibited the increases in the levels of the pro-inflammatory cytokines IL-1β and TNF-α. The T helper 17 (Th17)/regulatory T (Treg) cell balance was restored by decreasing the expression of IL-17A and IL-6 in intestinal tissues, increasing the expression of TGF-β, and decreasing the number of Th17 cells in each EBN dose group. These findings suggest that EBN has a protective effect on DSS-mediated colitis in mice, mainly by restoring the Th17/Treg cell balance.

## Introduction

Ulcerative colitis (UC) is a major form of inflammatory bowel disease (IBD) characterized by recurring and diffuse inflammation in the rectal and colonic mucosa. The incidence and prevalence rates of UC in North America and northern Europe are higher than those in Asia, although there is an increasing incidence in Asia (Ordás et al., 2012; Feuerstein et al., 2019). As an important public health problem and a global disease, UC tends to affect young people, which could increase the risk of colorectal cancer (Feuerstein et al., 2019). Risk factors for the development of UC are related to disruption of the intestinal mucosal barriers, which can be caused by infections, medications or familial genetics. Chronic and relapsing immune responses and clinical symptoms seriously reduce the quality of life of patients with UC. As a result of its complicated pathogenesis, UC has not yet been cured, and the goal of therapy in UC is to first induce clinical remission and then achieve steroid-free maintenance of remission (Feuerstein et al., 2019). In addition, due to its structure and long-term treatment, sulfasalazine (SASP), one of common used for UC, could cause some serious adverse reactions such as infertility, nephrotoxicity and hepatotoxicity (Linares et al., 2011). Therefore, there is an urgent need to develop novel therapeutic approaches for UC patient treatment.

Dysregulation of immune responses is considered an important factor in the development of UC. Recent research has shown that the immune response and inflammatory pathway of UC are driven by dynamic and complex interactions of cells and cytokines (Tatiya-Aphiradee et al., 2018). Moreover, T helper (Th) 17 cells and regulatory T (Treg) cells play crucial roles in UC pathogenesis by regulating, suppressing and maintaining inflammation (Tatiya-Aphiradee et al., 2018). Additionally, the homoeostatic balance between Th17 cells and Treg cells is disturbed in the mucosa of patients with UC (Hartog et al., 2015). Moreover, the balance between Th17 cells and Treg cells has a significant role in the T cell-mediated immune response in the intestine, and this balance is a novel and potential therapeutic target in UC (Bettelli et al., 2006; Xu et al., 2007; Lee et al., 2018).

Edible bird’s nest (EBN; known as Yanwo in Chinese), which comes from swiftlets belonging to the family *Apodidae* and the genus *Aerodramus*, has multiple nutritional and pharmaceutical benefits, such as pro-conception effects, neuroprotective effects and bone loss improvement (Matsukawa et al., 2011; Careena et al., 2018; Albishtue et al., 2019; Hou et al., 2019). Additionally, some studies have shown that EBN with antiviral activities can regulate the expression of some immune cytokines involved in influenza A virus infection, such as tumour necrosis factor α (TNF-α), interferon (IFN)-γ, interleukin (IL)-1β, IL-6, IL-10 and chemokine (C-C motif) ligand (CCL) 2 (Haghani et al., 2016; Haghani et al., 2017). Several pro-inflammatory cytokines (e.g., IL-1β, IL-6 and TNF-α) that can induce Th17 cell polarization have key roles in the pathogenesis of UC (Acosta-Rodriguez et al., 2007; Gálvez, 2014; Lee et al., 2018). In addition, IL-10 is an anti-inflammatory cytokine that is involved in Treg cell differentiation, leading to aberrant Treg cell function and increased susceptibility to UC (Boden and Snapper, 2008; Achour et al., 2017).

However, the protective effect of EBN in the context of UC is still unclear. Based on the theories mentioned above, we hypothesized that EBN could ameliorate the severity of UC by regulating pro-inflammatory and anti-inflammatory cytokines to maintain the Th17/Treg cell balance. In this study, we investigated the effect and mechanism of EBN in a dextran sulfate sodium (DSS)-induced UC model, which could provide a reference for drug development for UC therapy.

## Materials and Methods

### Animals and groupings

**FIGURE 1:**
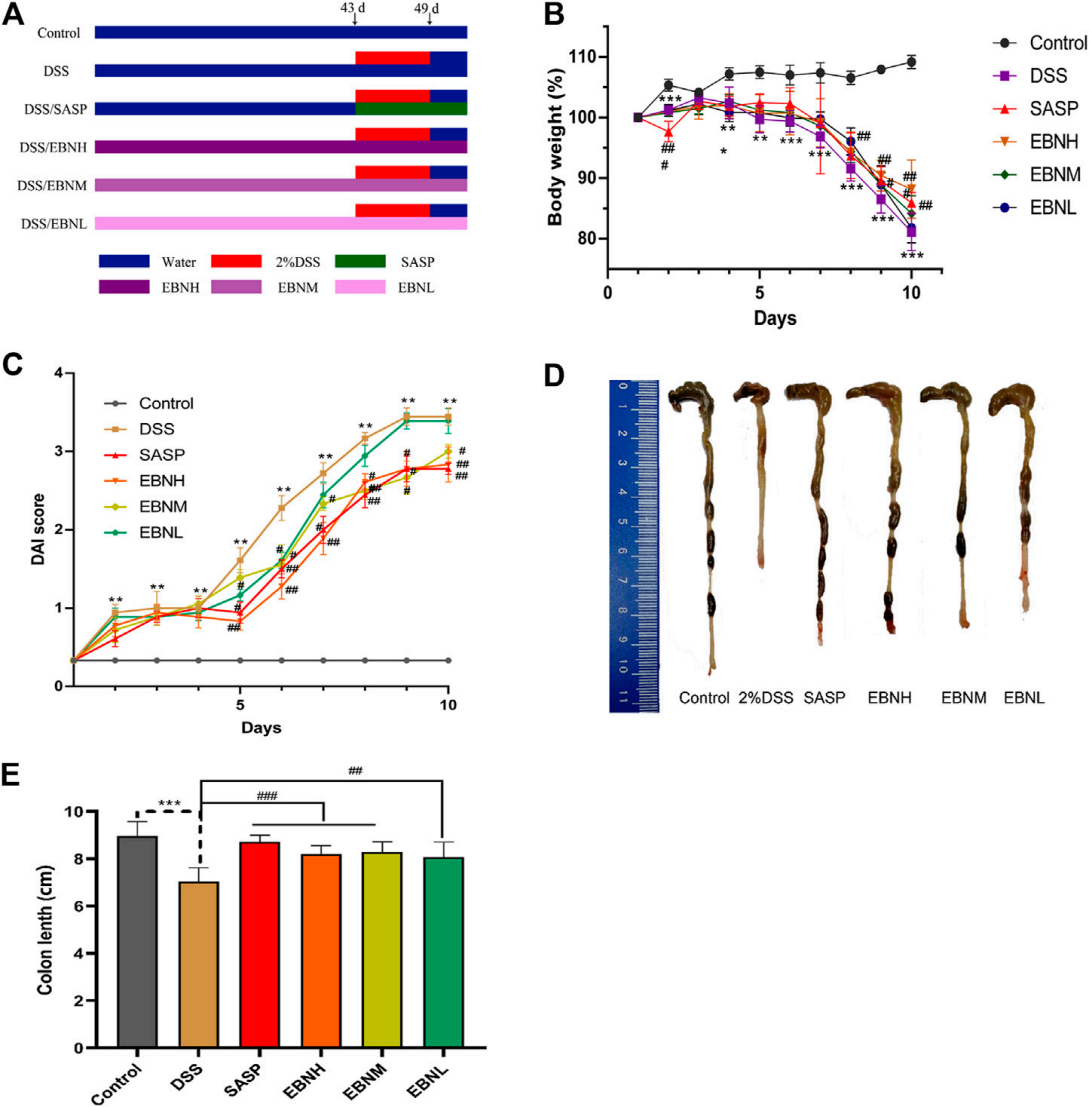
Effects of EBN pretreatment on the clinical symptoms of DSS-induced colitis in C57BL/6J mice. **(A)** Schematic showing the experimental dosing design. **(B)** Effect of EBN on body weight during the modeling period of colitis induced by DSS. **(C)** Effect of EBN on the disease activity index (DAI) of colitis induced by DSS. **(D,E)** Effect of EBN on colon length in mice with colitis induced by DSS. **P < 0.05 and ***P < 0.001 compared with the control group; #*P* < 0.05, ##*P* < 0.01 and ###*P* < 0.001 compared to the DSS group ($\bar{x}\pm S$, *n* = 6).

Male C57BL/6J mice (6°weeks old) were obtained from the animal laboratory of Guangzhou University of Chinese Medicine (production license No. SCXK [YUE]-2008–0034). All the experimental procedures were approved by the International Institute for Translational Chinese Medicine Animal Care and Use Committee, Guangzhou University of Chinese Medicine. Animals were housed in a well-ventilated room maintained at a temperature of 22–26°C with 12°h light/dark cycles and 40–70% humidity. Then, animals were randomly divided into six groups, each consisting of six animals, including the control group, model group (DSS, Yeasen biotech Co., Ltd., Shanghai, China), sulfasalazine (SASP, Shanghai Zhongxi Sunve Pharmaceutical Co., Ltd., Shanghai, China) group, low-dose EBN group (EBNL, 0.3 g/kg), moderate-dose EBN group (EBNM, 0.7 g/kg) and high-dose EBN group (EBNH, 1.3 g/kg). UC was induced in mice by administering 5°ml 2% DSS daily for 7°days; the animals in the control group were administered distilled water only. Each time point of drug administration is shown in Figure 1A. Body weight was measured once a week from the first week to the sixth week. Additionally, the body weight, faecal morphology and faecal occult blood of mice were recorded every day after the modelling period. At 53°days, the mice were sacrificed by cervical dislocation, and then colon specimens (from the caecum to the anal terminus the colon) and spleen specimens were collected.

### Preparation of Edible Bird’s Nest Extracts

EBN was provided by XIAMEN YAN PALACE SEELONG FOOD Co., Ltd (XIAMEN YAN PALACE SEELONG FOOD Co., Ltd., Xiamen, China) and was identified as the saliva of *Apodidae Aerodramus fuciphagus*. A voucher specimen was dried at 4°C and stored in an airtight container in the Laboratory Animal Center, Guangzhou University of Chinese Medicine. Briefly, the ground EBN was immersed in distilled water for an hour and boiled at 100°C for 30 min. The extracts were stored at 4°C for further use.

### Protein Identification of Edible Bird’s Nest Extracts by High Performance Liquid Chromatography/Electrospray Ionization

Characterization of sialic acid in EBN extracts was detected by HPLC (Easy-nLC1000). Chromatographic separation was achieved on a 3 μm Thermo scientific EASY column C18 column (75 μm × 100 mm), with column temperature maintained at 25°C. The mobile phases consisted of 0.1% formic acid solution (A) and acetonitrile formate in aqueous solution (B) using a gradient elution. The flow rate was 250 nL/min. The mass spectrometer was operated in electrospray ionization (ESI). The full scan setting parameters are as follows: ion polarity: positive ion; parent ion scanning range, 300–1800 m/z; first order mass resolution, 70,000 at m/z 200; Secondary mass resolution, 17,500 at m/z 200; ionspray voltage, 2 kV; capillary temperature, 250°C; collision energy, 27%HCD. The sialic acid was identified by the retention time and quantified by comparison of integrated peak areas with a known amount of sialic acid-labelled C18 standard sample.

### Evaluation of Ulcerative Colitis Severity Based on Disease Activity Index Scores

The disease activity index (DAI) has been widely used to assess the severity of UC in animal models in light of the study of Wirtz et al. (2017), Zhu et al. (2019). Briefly, DAI scores are calculated as the mean of the total scores of all parameters including weight loss, stool consistency and the degree of intestinal bleeding. The degree of intestinal bleeding was also assessed by a qualitative detection kit for faecal occult blood (Leagene Biotechnology Co., Ltd., Beijing, China). The criteria of the scoring system are shown in Table 1.

**TABLE 1:** Scoring system for calculating DAI scores.

Score	Weight loss	Stool consistency	Blood
0	None	Normal	Negative haemoccult
1	1–5%	Soft but still formed	1+
2	6–10%	Soft	2+
3	11–18%	Very soft; wet	3+
4	>18%	Watery diarrhoea	Blood traces in stool visible

### Morphological and Histological Evaluation

Colonic tissues were embedded in paraffin and stained with haematoxylin and eosin (HE). Then, we assessed the inflammation-associated histological changes in the colon based on lamina propria inflammatory cell infiltration, and the morphology of the intestinal epithelium and glandular and goblet cells.

### Myeloperoxidase Activity Assay

Radioimmunoprecipitation assay (RIPA) lysis buffer (BIOSYNTHESIS BIOTECHNOLOGY Co., Ltd., Beijing, China) was used to lyse tissues, and 10 mg tissue was obtained for each sample. Following washing with cold PBS, the tissues were resuspended in 9 volumes of PBS (included in the myeloperoxidase Activity Assay Kit) and then centrifuged at 4°C and 2,500 r/min for 10 min. The supernatant was collected and transferred to a clean tube, which was then placed on ice. Myeloperoxidase (MPO) activity was assayed using the myeloperoxidase Activity Assay Kit (Abcam Co., Ltd., Cambridge, United States) with measurement of the absorbance of the sample at 460 nm using a microplate reader. The specific MPO activity in the colon was measured as units/g (U/g) protein.

### Cytokine Analysis by Enzyme-Linked Immunosorbent Assay

Colonic tissues were soaked in 5 volumes of 1X PBS containing 1% phenylmethanesulfonyl fluoride (PMSF). Then, the tissues were placed on a tissue grinder and ground at 10 Hz for 1 min. Next, the specimens were lysed at 4°C for 30 min and then centrifuged at 4°C and 12,000 r/min for 10 min. The supernatant was collected and transferred to a clean tube. Subsequently, we measured the levels of IL-1β, TNF-α, IL-10, IL-17, IL-6 and TGF-β in the colonic tissues using ELISA kits according to the manufacturer's instructions (MultiSciences Biotech Co., Ltd., Hangzhou, China).

### Preparation of Splenic Single-Cell Suspensions

Spleens were placed in 6 mm sterile petri dishes and mechanically dissociated. Following filtration through a 100 mesh cell filter, we obtained splenic cell suspensions. Then, the suspensions were centrifuged at 4°C and 1,500 r/min for 5 min to obtain the cell pellets. The pellets were resuspended in 2 ml PBS and then centrifuged at 4°C and 1,500 r/min for 5 min; the supernatant was discarded. Subsequently, RPMI 1640 medium containing 10% PBS was used to resuspend the pellets, which were then counted, and the cell concentration was adjusted to 1 × 10^6^/ml.

### Measurement of the Proportions of CD4^+^IL-17A^+^ Th17 Cells and CD25^+^Foxp3^+^ Treg Cells by Flow Cytometry

Th17 and Treg cells were isolated from mouse spleen tissues as cell suspensions and evaluated by flow cytometry. The Th17 cells were positively sorted by CD4 and IL-17A. We used FACS analyses and gated on live CD4^high^ cells and IL-17A^high^ cells. CD4^high^/IL-17A^high^ double-positive cells were used to determine the percentages of Th17 cells. The Treg cells were positively sorted by CD25 and Foxp3. We used FACS analyses and gated on live CD25-high cells and Foxp3-high cells. CD25-high/Foxp3-high double-positive cells were used to determine the percentages of Treg cells.

### Measurement of the Expression of IL-17A and Foxp3 in Colonic Tissues by Immunohistochemistry

Paraffin sections of colonic tissues were dewaxed; soaked in xylene I, II and III for 15 min each; and hydrated with ethanol (anhydrous ethanol I, anhydrous ethanol II, 85% ethanol and 75% ethanol for 5 min each) and distilled water. After performing antigen repair and blocking endogenous peroxidase activity, the sections were blocked in goat serum at room temperature for 30 min. Then, the sections were washed three times in PBS for 5 min each time and blocked with 3% bovine serum albumin. After blocking, the sections were incubated with anti-IL-17A and anti-Foxp3 (eBioscience Co., Ltd., CA, United States) antibodies overnight at 4°C. The sections were washed 3 times in PBS and incubated with secondary antibodies (EARTH Co., Ltd., CA, United States) in an incubator at room temperature for 50 min. The tissue sections were incubated with a diaminobenzidine substrate solution (BOSTER Co., Ltd., Wuhan, China).

### Measurement of the Expression of IL-17A and Foxp3 by Western Blotting

Colonic tissues were lysed in RIPA buffer (Sigma-Aldrich) containing a PMSF protease inhibitor. The specimens were placed in a Tissue Lyser at 70 Hz for 60 s. The homogenate was lysed on ice for 30 min and centrifuged at 12,000 r/min for 10 min at 4°C. Protein concentrations were determined using a Bradford protein assay kit (Beyotime Co., Ltd., Shanghai, China). The samples were boiled for 5 min in loading buffer. The proteins were transferred to nitrocellulose membranes with 0.45 μm-diameter pores. The membranes were blocked with 5% non-fat dry milk for 1 h at room temperature and then incubated with primary antibodies, including anti-IL-17A and anti-Foxp3 (eBioscience Co., Ltd., CA, United States). After incubation overnight, the membranes were washed 3 times for 10 min each time in PBST and incubated with a secondary antibody (Cell Signaling Technology) for 1 h at room temperature. The protein bands were visualized with Immobilon Western HRP Substrate (Millipore Co., Ltd., MA, United States). Densitometric analyses of the bands were performed using ImageJ software. All data are representative of at least 3 independent experiments.

### Statistical Analysis

Results were analyzed with SPSS 22.0 software and are expressed as the mean ± standard deviation. Mauchly’s test of sphericity was used to analyse repeated measurement data. If the repeated measurement data did not conform to Mauchly’s test of sphericity (*p* < 0.05), the multivariate test Roy’s maximum root was used to identify statistically significant differences (*p* < 0.05). Data considered to conform to a normal distribution (*p*-value of the Shapiro-Wilk test > 0.05) were analysed by one-way analysis of variance (ANOVA). Data that did not conform to a normal distribution were analysed by nonparametric tests and are shown as quartiles (M [*P*25–75]). *P* < 0.05, *P* < 0.01 and *P* < 0.001 were considered significant. GraphPad Prism 8.0 software was used to draw images.

## Results

### Characterization of Edible Bird’s Nest Extracts

**FIGURE 2:**
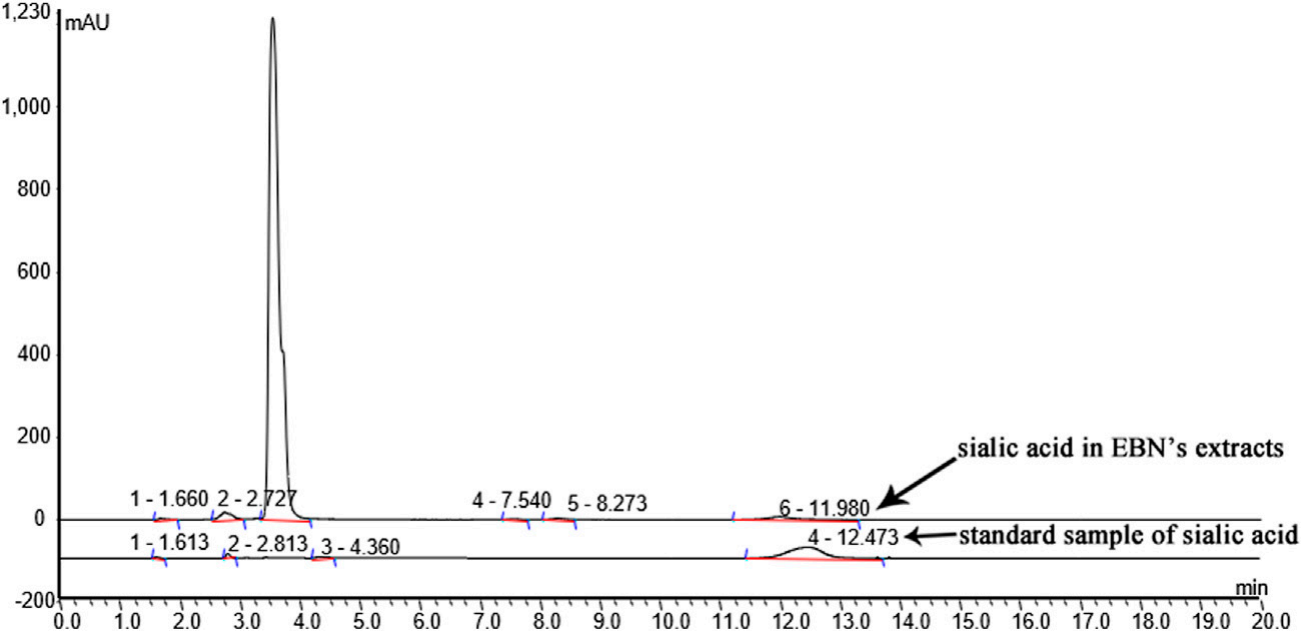
Characterization of EBN extracts.

To identify the sialic acid in EBN extracts, we analyzed the EBN extracts using HPLC/ESI. The polypeptides of EBN were isolated and purified by HPLC. Then ESI formed multi-charge ions, which can be used to detect polypeptide molecules in a small M/Z range, the result was shown in Figure 2.

### Effects of Edible Bird’s Nest Pretreatment on the Clinical Symptoms of Dextran Sulfate Sodium-Induced Colitis in C57BL/6J Mice

First, we evaluated the efficacy of EBN pretreatment in alleviating the clinical symptoms of UC. All mice exhibited normal activity, diet, stool consistency and weight gain before modelling. In comparison with those in the control group, the mice in the model group showed not only observably decreased body weight (Figure 1B) and colon length (Figures 1D,E) but also observably increased DAI scores (Figure 1C, Supplementary Tables S1–S6). As shown in Figure 1C, the DAI scores of the SASP group and EBN groups began to show relief of disease symptoms in mice on the 5th day after modelling, and the DAI scores of these groups were significantly lower than those of the model group. In addition, the macroscopic morphology of colonic tissues showed colon oedema, a reduced caecal volume and an enteric cavity with bloody stools. Nevertheless, the mice pretreated with EBN exhibited reversed colonic shortening and relieved colonic oedema. These results suggest that EBN can alleviate the clinical symptoms of UC and could be a latent drug for UC.

### Effects of Edible Bird’s Nest Pretreatment on Inflammation in Dextran Sulfate Sodium-Induced Ulcerative Colitis

**FIGURE 3:**
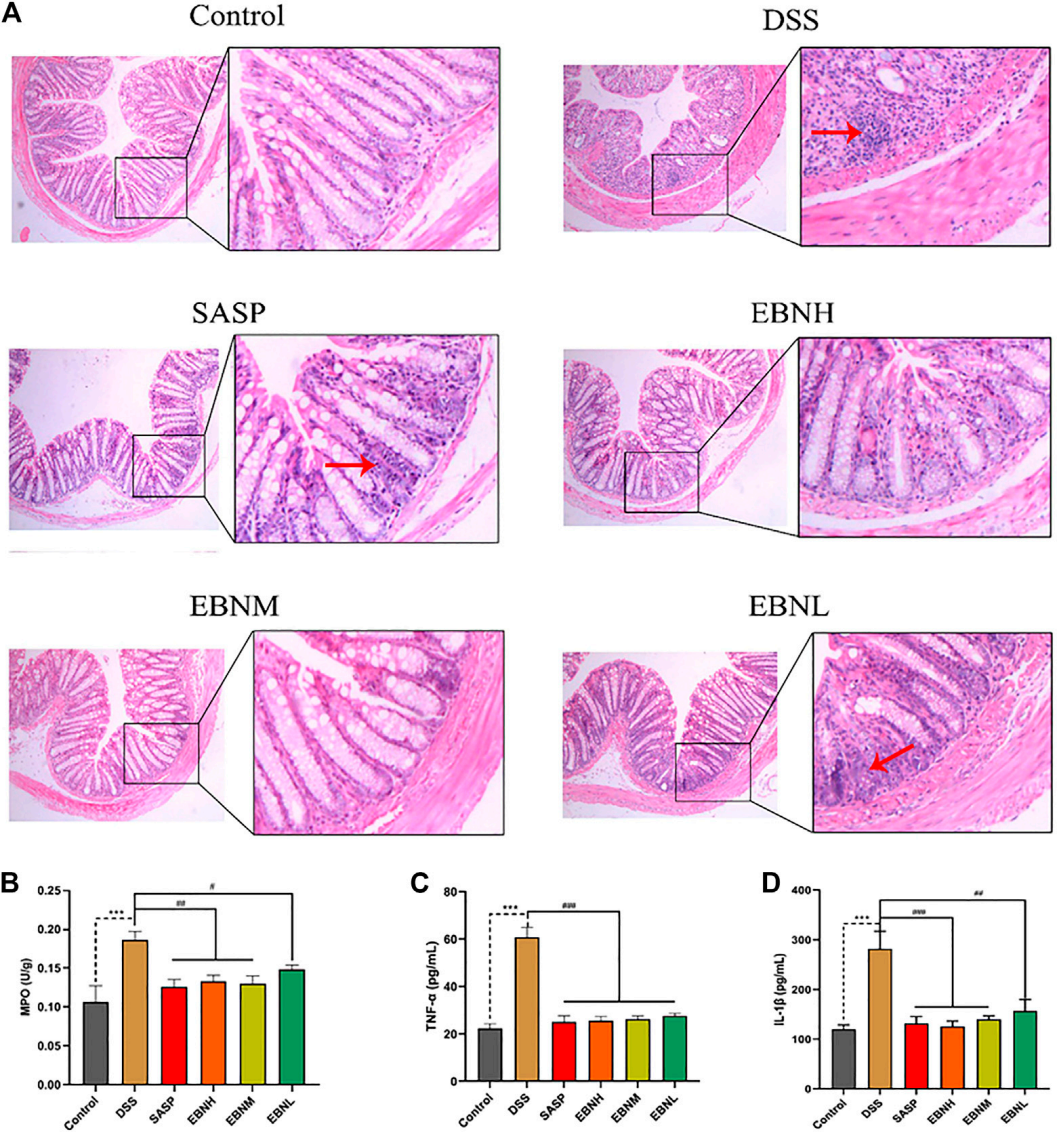
Effects of EBN pretreatment on inflammation in DSS-induced UC. **(A)** Effect of EBN on colonic histomorphology in mice with colitis induced by DSS (50×). **(B–D)** Effects of EBN on MPO activity and TNF-α and IL-1β levels in colonic tissues of mice with colitis induced by DSS. **P < 0.01 and ***P < 0.001 compared with the control group, #*P* < 0.05 and ##*P* < 0.01 compared to the DSS group ($\bar{x}\pm S$, *n* = 6).

To evaluate the extent of damage and inflammation in colonic tissues, histological analysis was performed. As shown in Figure 3A, HE staining of tissues from the control group showed an intact colonic structure with aligned crypts and glands and no inflammatory cell infiltration. Consistent with previous studies, the colonic tissues of the model group showed extensive inflammatory cell infiltration in the submucosa, a large area of exfoliated intestinal epithelium, jumbled arrangement of the glands, reduced goblet cell numbers and oedema in the submucosa (Wirtz et al., 2017; Zhu et al., 2019). In contrast, the mice pretreated with EBN showed a low level of inflammation and a more complete structure of the submucosa.

In addition, we detected MPO activity and cytokine levels in colonic tissues, which can reflect the severity of inflammation. As shown in Figures 3A,B higher level of MPO activity was found in the model group than in the control group. However, this activity was observably reduced by treatment with EBN or SASP. On the other hand, the EBN-pretreated groups showed reductions in the expression of TNF-α and IL-6 compared with the model group (Figures 3C,D). These results also demonstrated that EBN inhibited the inflammation induced by DSS by reducing the levels of pro-inflammatory cytokines.

### Effects of Edible Bird’s Nest Pretreatment on the Expression of Th17 and Treg Cell-Associated Cytokines in Colonic Tissues

**FIGURE 4:**
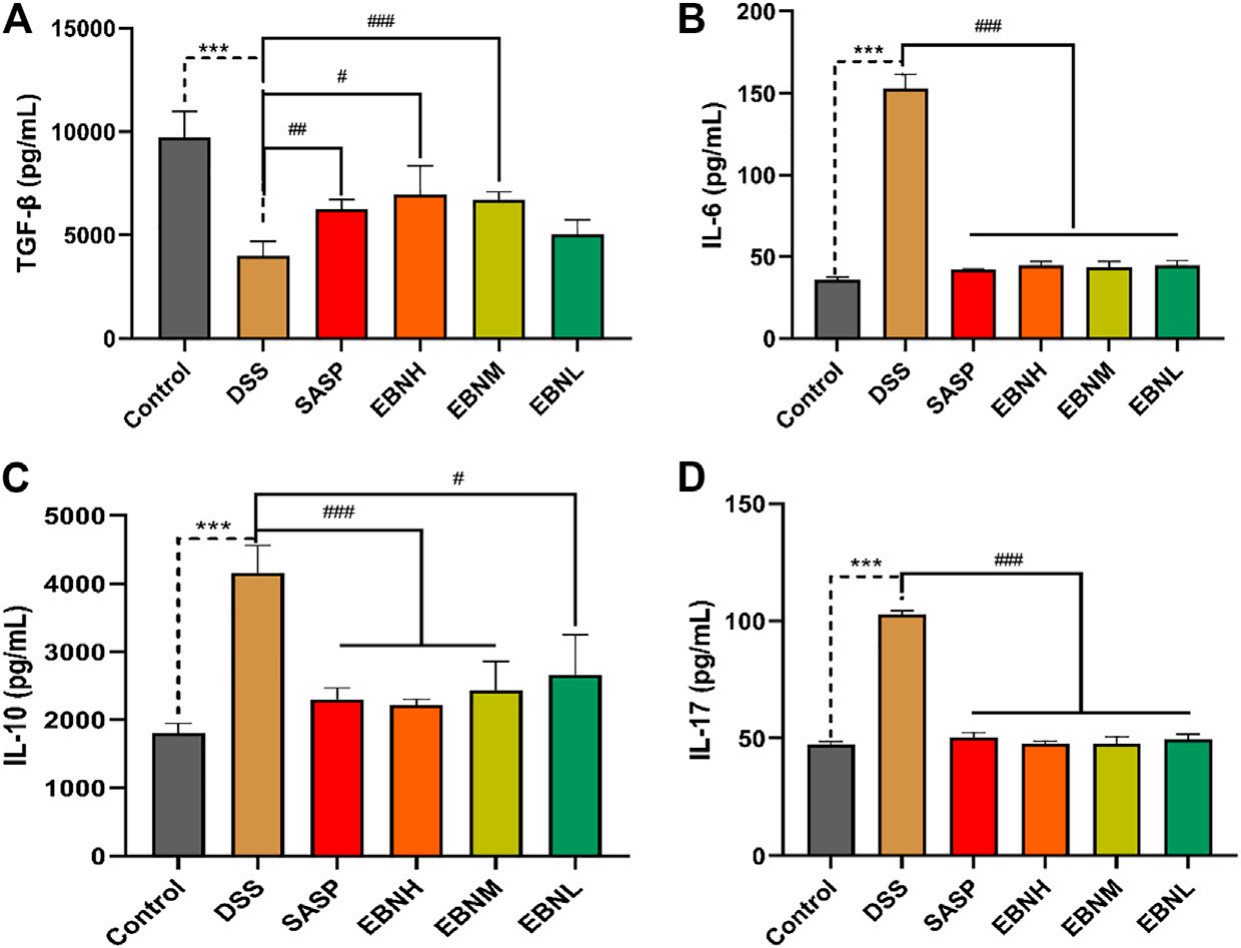
Effects of EBN pretreatment on the expression of Th17 and Treg cell-associated cytokines in colonic tissues. **(A–D)** Effects of EBN on the expression of TGF-β, IL-6, IL-10 and IL-17 in colonic tissues. ***P < 0.001 compared with the control group; #*P* < 0.05, ##*P* < 0.01 and ###*P* < 0.001 compared to the DSS group ($\bar{x}\pm S$, *n* = 6).

The effects of EBN on DDS-induced Th17 and Treg cell-associated cytokines were next explored. As shown in Figure 4, the level of TGF-β secreted by Treg cells was decreased in the colon of DSS-induced C57BL/6J mice compared to that of control group mice, while the levels of IL-6 and IL-17 were upregulated. In contrast, in comparison with those in the model group, the mice pretreated with EBN showed lower levels of IL-6 and IL-17, as well as a higher level of TGF-β. These results suggest that EBN ameliorates the inflammatory response induced by DSS by regulating the secretion of Th17 and Treg cell-associated cytokines.

### Effects of Edible Bird’s Nest Pretreatment on the Balance of Th17/Treg Cells

**FIGURE 5:**
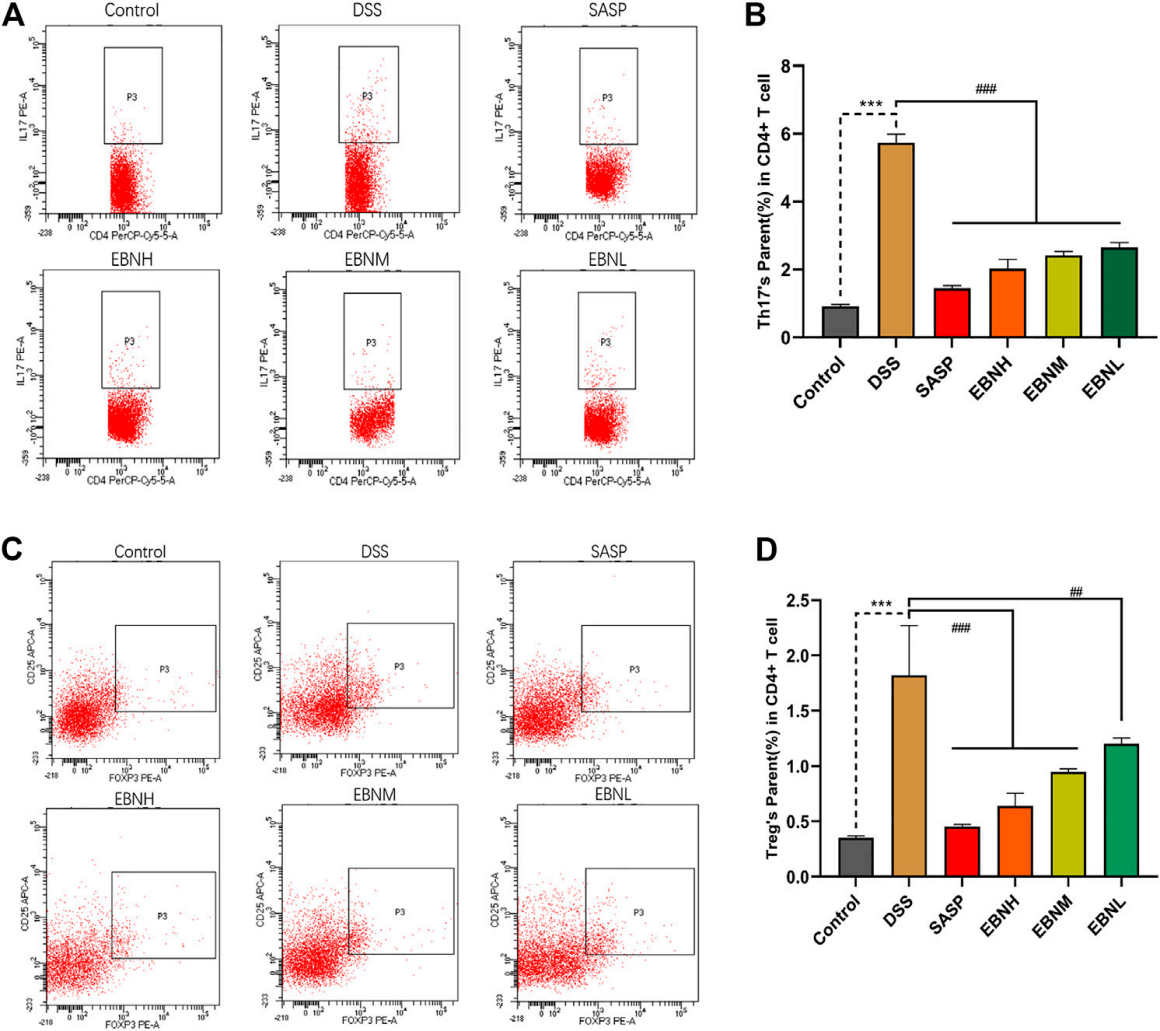
Effects of EBN pretreatment on the proportions of Th17 cells and Treg cells in the CD4^+^ T cell compartment of the spleen. **(A,B)** Effect of EBN on the proportion of CD4^+^IL-17A^+^ Th17 cells in the spleen of mice with colitis. **(C,D)** Effect of EBN on the proportion of CD25^+^Foxp3^+^ Treg cells in the spleen of mice with colitis. ***P < 0.001 compared with the control group; #*P* < 0.05, ##*P* < 0.01 and ###*P* < 0.001 compared to the DSS group ($\bar{x}\pm S$, *n* = 3).

To explore the effect of EBN on the immune system in DSS-induced UC, the proportions of Th17 cells and Treg cells in the CD4^+^ T cell compartment of the spleen were measured by flow cytometry. As shown in Figure 5, treatment with DSS increased the proportions of Th17 cells and Treg cells. However, the proportions of Th17 cells and Treg cells were both restored in all EBN pretreatment groups.

**FIGURE 6:**
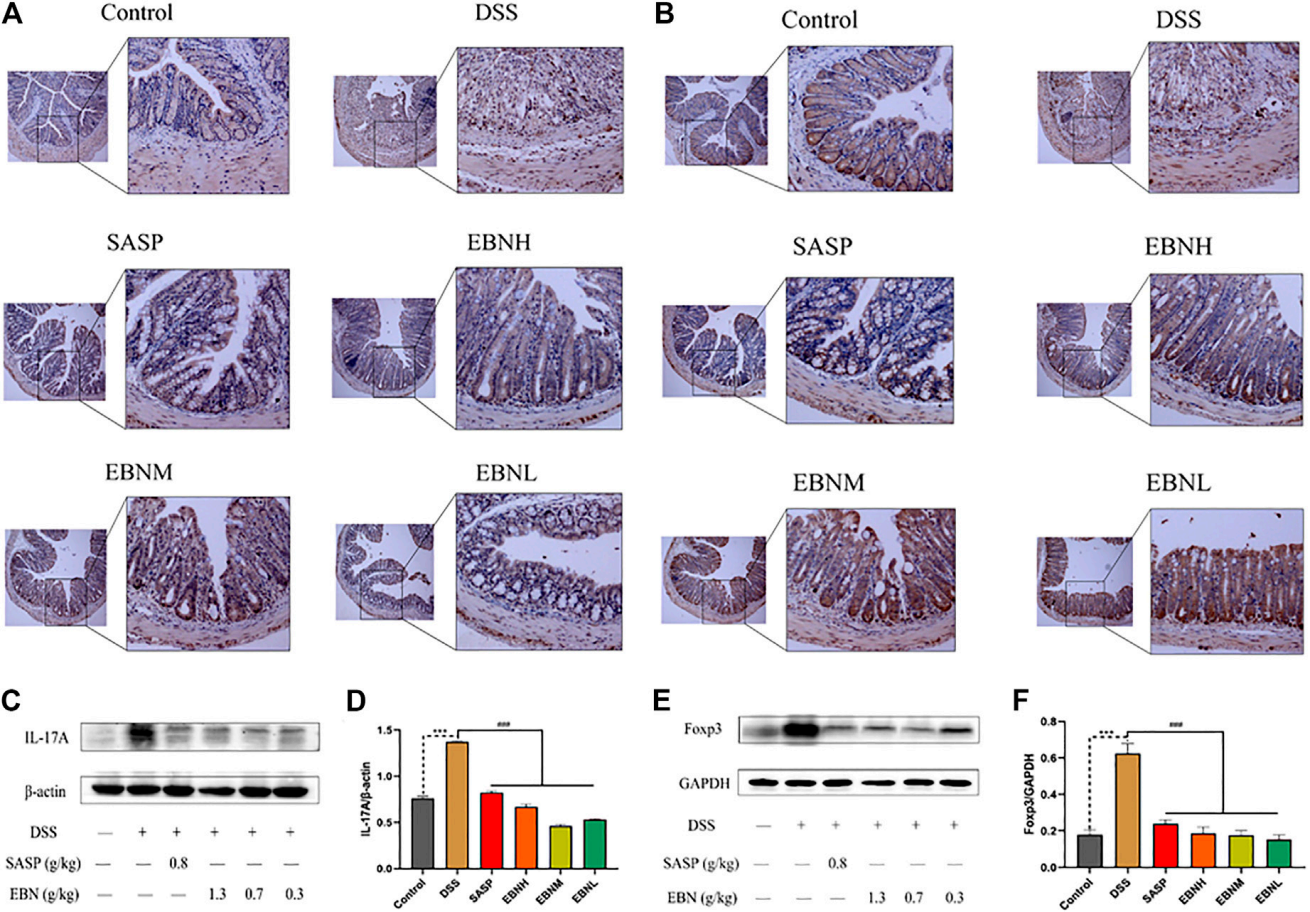
Effects of EBN pretreatment on the expression of Th17 and Treg cell-associated proteins in colonic tissues. **(A,B)** Effects of EBN on the expression of IL-17A and Foxp3 in colonic tissues detected by IHC (100×). **(C,D)** Effect of EBN on IL-17A expression in colonic tissues of mice with colitis. **(E,F)** Effect of EBN on Foxp3 expression in colonic tissues of mice with colitis. ***P < 0.001 compared with the control group; #*P* < 0.05, ##*P* < 0.01 and ###*P* < 0.001 compared to the DSS group ($\bar{x}\pm S$, *n* = 6).

We further explored the effects of EBN pretreatment on the expression of Th17 and Treg cell-associated proteins in colonic tissues. Immunohistochemistry (IHC) and western blot results showed that the expression of IL-17A and Foxp3 in the model group was significantly higher than that in the control group (Figure 6). However, in comparison with modelling alone, EBN pretreatment dramatically inhibited the overexpression of IL-17A and Foxp3 induced by DSS (Figure 6). These results indicated that the counts of Th17 cells and Treg cells in the colon could be restored to a normal balance, which was consistent with the results for the spleen. These findings suggest that EBN can help the immune system reverse the overreaction in UC by regulating the balance of Th17/Treg cells.

## Discussion

EBN, a natural product with value as both a medicine and a food, has been used to prevent and treat diseases in the Chinese population since the Yuan dynasty (Fan. et al., 2020). Recent studies have found that EBN can potentiate mitogenic responses, increase the lifespan, protect neurons, promote bone mineralization, increase bone density, inhibit influenza virus, enhance the antioxidant capacity, alleviate inflammation, promote B cell proliferation and activation and reduce intestinal immune injury (Roh et al., 2012; Yew et al., 2014; Hu et al., 2016; Zhao et al., 2016; Quek et al., 2018; Hou et al., 2019). Moreover, EBN restores the immune system in the context of infection by influencing viruses through regulation of pro-inflammatory cytokines, such as IL-6 and TNF-α, and cytokines with regulatory properties, such as IL-10 and CCL2, which are also involved in the progression of UC (Haghani et al., 2016; Lee et al., 2018). Hence, we thoroughly examined the effects of EBN on DSS-induced UC and the associated mechanism in C57BL/6J mice.

Consistent with previous studies, DSS-induced C57BL/6J mice showed a series of symptoms of UC, such as weight loss, diarrhoea, haematochezia, abbreviated colon length, relatively high DAI scores and inflammatory cell infiltration in colonic tissues (Figures 1, 3) (Wirtz et al., 2017; Cao et al., 2018; Yan et al., 2018).

MPO is a recognized biomarker of tissue inflammation and can catalyse the production of potent reactive oxygen species (ROS). Additionally, MPO activity perpetuates inflammation and contributes to host tissue injury in patients with IBD (Chami et al., 2018). In experimental colitis, disease severity is often positively correlated with the levels of MPO activity and pro-inflammatory cytokines, such as TNF-α and IL-1β (Kim et al., 2012). Our study showed that EBN pretreatment significantly reduced MPO activity and the expression of TNF-α and IL-1β in the colon in a DSS-induced UC model. These results indicated that EBN pretreatment could resist inflammation and protect colonic tissues by reducing the infiltration of neutrophils and levels of pro-inflammatory cytokines.

Three unique components including a thick luminal mucin layer, a single epithelial cell barrier, and secretion of anti-inflammatory factors can protect the gut mucosal lamina propria from luminal resident microbiota (Ueno et al., 2018). In particular, pro-inflammatory and anti-inflammatory cytokines are crucial for the development of T cells (Ueno et al., 2018). Moreover, the imbalance between Th17 cells and Treg cells substantially contributes to the intestinal immune disturbance and subsequent tissue injury in UC (Xu et al., 2017). Moreover, IL-6 can induce the proliferation of Th17 cells, which can secrete pro-inflammatory cytokines, such as IL-17 and TNF-α, to play pathogenic roles in intestinal inflammation (Kimura and Kishimoto, 2010; Lee et al., 2018). Our results indicated that EBN reduced the infiltration of neutrophils and levels of pro-inflammatory cytokines by inhibiting the proliferation of Th17 cells.

However, several studies have shown opposite results. A clinical study showed a relatively high level of Th17 cells and relatively low level of Treg cells in UC patients (Gong et al., 2016). In addition, relatively high levels of CD4^+^ Treg cells and IL-10 but little TGF-β has been observed in inflamed sites in the gastric mucosa (Kindlund et al., 2017; Ma et al., 2018). Consistent with previous studies, the proportion of Treg cells in the CD4^+^ T cell compartment of the spleen was increased by DSS, which could lead to a higher level of IL-10 to limit inflammation. Treg cell levels were increased in the spleen and colon as a negative feedback regulation mechanism of the body in response to a sharp rise in the Th17 cell proportion (Kindlund et al., 2017). Because of the immunomodulatory activity of EBN, the mice in the EBN groups with a state of mild inflammation could inhibit the development of Treg cells by reducing the negative feedback regulation in response to the low level of Th17 cells. Therefore, we inferred that the primary targets of EBN, which could ameliorate DSS-induced UC and restore the Th17/Treg cell balance, are Th17 cells.

## Conclusion

Our results have shown for the first time that in mice with UC, EBN improves symptoms of UC, reduces colonic injury, and inhibits the increases in the levels of the pro-inflammatory cytokines IL-1β and TNF-α. The Th17/Treg cell balance was restored by decreasing the expression of IL-17A and IL-6 in intestinal tissues and decreasing the proportion of Th17 cells in each EBN dose group. Additionally, our results suggest that EBN has a protective effect on DSS-mediated UC mice, mainly by inhibiting the development and secretory function of Th17 cells. Also, the different efficacy among EBNL group, EBNM group and EBNH group were not statistically significant. Thus, we suggested that the optimal concentration of EBN to treat UC mice is 0.3 g/kg. However, pathological changes in UC include damage to the intestinal epithelium and changes in intestinal mucosal permeability and tight junction proteins. Therefore, whether EBN also protects the intestinal barrier to ameliorate UC by improving intestinal permeability and tight junction protein expression remains unclear and deserves further study. In conclusion, these results provide further evidence for the use of EBN as a treatment for the prevention of UC.

## Data Availability

The raw data supporting the conclusions of this article will be made available by the authors, without undue reservation.
