# Supplementary material for: Edible Bird’s Nest Ameliorates Dextran Sulfate Sodium-Induced Ulcerative Colitis in C57BL/6J Mice by Restoring the Th17/Treg Cell Balance
Source: Front Pharmacol. 2021 Apr 21;12:632602. doi: 10.3389/fphar.2021.632602 (PMC8097138; doi:10.3389/fphar.2021.632602)
Supplement: Supplementary file 1 [file datasheet1.docx]

Supplementary Material

# Supplementary Tables

Supplementary Table 1. The original data of each indicator of DAI scores in control group

| Day | Control Group | | | | | | | | | | | | | | | | | | | | | | | | | | | | | |
| --- | --- | --- | --- | --- | --- | --- | --- | --- | --- | --- | --- | --- | --- | --- | --- | --- | --- | --- | --- | --- | --- | --- | --- | --- | --- | --- | --- | --- | --- | --- |
|  | 1 | | | | | 2 | | | | | 3 | | | | | 4 | | | | | 5 | | | | | 6 | | | | |
|  | Weight loss | Stool consistency | Blood | Sum | DAI scores | Weight loss | Stool consistency | Blood | Sum | DAI scores | Weight loss | Stool consistency | Blood | Sum | DAI scores | Weight loss | Stool consistency | Blood | Sum | DAI scores | Weight loss | Stool consistency | Blood | Sum | DAI scores | Weight loss | Stool consistency | Blood | Sum | DAI scores |
| 1 | 0 | 1 | 0 | 1 | 0.333333 | 0 | 1 | 0 | 1 | 0.333333 | 0 | 1 | 0 | 1 | 0.333333 | 0 | 1 | 0 | 1 | 0.333333 | 0 | 1 | 0 | 1 | 0.333333 | 0 | 1 | 0 | 1 | 0.333333 |
| 2 | 0 | 1 | 0 | 1 | 0.333333 | 0 | 1 | 0 | 1 | 0.333333 | 0 | 1 | 0 | 1 | 0.333333 | 0 | 1 | 0 | 1 | 0.333333 | 0 | 1 | 0 | 1 | 0.333333 | 0 | 1 | 0 | 1 | 0.333333 |
| 3 | 0 | 1 | 0 | 1 | 0.333333 | 0 | 1 | 0 | 1 | 0.333333 | 0 | 1 | 0 | 1 | 0.333333 | 0 | 1 | 0 | 1 | 0.333333 | 0 | 1 | 0 | 1 | 0.333333 | 0 | 1 | 0 | 1 | 0.333333 |
| 4 | 0 | 1 | 0 | 1 | 0.333333 | 0 | 1 | 0 | 1 | 0.333333 | 0 | 1 | 0 | 1 | 0.333333 | 0 | 1 | 0 | 1 | 0.333333 | 0 | 1 | 0 | 1 | 0.333333 | 0 | 1 | 0 | 1 | 0.333333 |
| 5 | 0 | 1 | 0 | 1 | 0.333333 | 0 | 1 | 0 | 1 | 0.333333 | 0 | 1 | 0 | 1 | 0.333333 | 0 | 1 | 0 | 1 | 0.333333 | 0 | 1 | 0 | 1 | 0.333333 | 0 | 1 | 0 | 1 | 0.333333 |
| 6 | 0 | 1 | 0 | 1 | 0.333333 | 0 | 1 | 0 | 1 | 0.333333 | 0 | 1 | 0 | 1 | 0.333333 | 0 | 1 | 0 | 1 | 0.333333 | 0 | 1 | 0 | 1 | 0.333333 | 0 | 1 | 0 | 1 | 0.333333 |
| 7 | 0 | 1 | 0 | 1 | 0.333333 | 0 | 1 | 0 | 1 | 0.333333 | 0 | 1 | 0 | 1 | 0.333333 | 0 | 1 | 0 | 1 | 0.333333 | 0 | 1 | 0 | 1 | 0.333333 | 0 | 1 | 0 | 1 | 0.333333 |
| 8 | 0 | 1 | 0 | 1 | 0.333333 | 0 | 1 | 0 | 1 | 0.333333 | 0 | 1 | 0 | 1 | 0.333333 | 0 | 1 | 0 | 1 | 0.333333 | 0 | 1 | 0 | 1 | 0.333333 | 0 | 1 | 0 | 1 | 0.333333 |
| 9 | 0 | 1 | 0 | 1 | 0.333333 | 0 | 1 | 0 | 1 | 0.333333 | 0 | 1 | 0 | 1 | 0.333333 | 0 | 1 | 0 | 1 | 0.333333 | 0 | 1 | 0 | 1 | 0.333333 | 0 | 1 | 0 | 1 | 0.333333 |
| 10 | 0 | 1 | 0 | 1 | 0.333333 | 0 | 1 | 0 | 1 | 0.333333 | 0 | 1 | 0 | 1 | 0.333333 | 0 | 1 | 0 | 1 | 0.333333 | 0 | 1 | 0 | 1 | 0.333333 | 0 | 1 | 0 | 1 | 0.333333 |

Supplementary Table 2. The original data of each indicator of DAI scores in DSS group

| Day | DSS Group | | | | | | | | | | | | | | | | | | | | | | | | | | | | | |
| --- | --- | --- | --- | --- | --- | --- | --- | --- | --- | --- | --- | --- | --- | --- | --- | --- | --- | --- | --- | --- | --- | --- | --- | --- | --- | --- | --- | --- | --- | --- |
|  | 1 | | | | | 2 | | | | | 3 | | | | | 4 | | | | | 5 | | | | | 6 | | | | |
|  | Weight loss | Stool consistency | Blood | Sum | DAI scores | Weight loss | Stool consistency | Blood | Sum | DAI scores | Weight loss | Stool consistency | Blood | Sum | DAI scores | Weight loss | Stool consistency | Blood | Sum | DAI scores | Weight loss | Stool consistency | Blood | Sum | DAI scores | Weight loss | Stool consistency | Blood | Sum | DAI scores |
| 1 | 0 | 1 | 0 | 1 | 0.333333 | 0 | 1 | 0 | 1 | 0.333333 | 0 | 1 | 0 | 1 | 0.333333 | 0 | 1 | 0 | 1 | 0.333333 | 0 | 1 | 0 | 1 | 0.333333 | 0 | 1 | 0 | 1 | 0.333333 |
| 2 | 0 | 2 | 1 | 3 | 1 | 0 | 1 | 2 | 3 | 1 | 0 | 2 | 1 | 3 | 1 | 0 | 1 | 1 | 2 | 0.666667 | 0 | 3 | 1 | 4 | 1.333333 | 0 | 1 | 1 | 2 | 0.666667 |
| 3 | 0 | 4 | 1 | 5 | 1.666667 | 0 | 1 | 1 | 2 | 0.666667 | 0 | 2 | 0 | 2 | 0.666667 | 0 | 3 | 2 | 5 | 1.666667 | 0 | 1 | 1 | 2 | 0.666667 | 0 | 1 | 1 | 2 | 0.666667 |
| 4 | 0 | 1 | 1 | 2 | 0.666667 | 0 | 2 | 0 | 2 | 0.666667 | 0 | 2 | 1 | 3 | 1 | 0 | 3 | 0 | 3 | 1 | 0 | 3 | 0 | 3 | 1 | 1 | 3 | 1 | 5 | 1.666667 |
| 5 | 1 | 3 | 2 | 6 | 1.666667 | 0 | 4 | 2 | 6 | 2 | 0 | 2 | 1 | 3 | 1 | 0 | 4 | 1 | 5 | 1.666667 | 1 | 2 | 1 | 4 | 1.333333 | 1 | 3 | 2 | 6 | 2 |
| 6 | 1 | 4 | 3 | 8 | 2.666667 | 1 | 4 | 2 | 7 | 2.333333 | 0 | 3 | 2 | 5 | 1.666667 | 0 | 4 | 3 | 7 | 2.333333 | 0 | 4 | 4 | 8 | 2.666667 | 1 | 3 | 2 | 6 | 2 |
| 7 | 1 | 3 | 4 | 8 | 2.666667 | 2 | 4 | 3 | 9 | 3 | 0 | 4 | 3 | 7 | 2.333333 | 1 | 4 | 4 | 9 | 3 | 1 | 4 | 2 | 7 | 2.333333 | 1 | 4 | 4 | 9 | 3 |
| 8 | 2 | 4 | 4 | 10 | 3.333333 | 3 | 4 | 3 | 10 | 3.333333 | 2 | 3 | 4 | 9 | 3 | 2 | 4 | 4 | 10 | 3.333333 | 2 | 4 | 3 | 9 | 3 | 2 | 4 | 3 | 9 | 3 |
| 9 | 3 | 4 | 2 | 9 | 3 | 3 | 4 | 3 | 10 | 3.333333 | 3 | 4 | 4 | 11 | 3.666667 | 3 | 4 | 4 | 11 | 3.666667 | 3 | 4 | 4 | 11 | 3.666667 | 3 | 4 | 3 | 10 | 3.333333 |
| 10 | 3 | 4 | 3 | 10 | 3.333333 | 4 | 3 | 3 | 10 | 3.333333 | 4 | 4 | 3 | 11 | 3.666667 | 3 | 4 | 2 | 9 | 3 | 4 | 4 | 3 | 11 | 3.666667 | 4 | 4 | 3 | 11 | 3.666667 |

Supplementary Table 3. The original data of each indicator of DAI scores in SASP group

| Day | SASP Group | | | | | | | | | | | | | | | | | | | | | | | | | | | | | |
| --- | --- | --- | --- | --- | --- | --- | --- | --- | --- | --- | --- | --- | --- | --- | --- | --- | --- | --- | --- | --- | --- | --- | --- | --- | --- | --- | --- | --- | --- | --- |
|  | 1 | | | | | 2 | | | | | 3 | | | | | 4 | | | | | 5 | | | | | 6 | | | | |
|  | Weight loss | Stool consistency | Blood | Sum | DAI scores | Weight loss | Stool consistency | Blood | Sum | DAI scores | Weight loss | Stool consistency | Blood | Sum | DAI scores | Weight loss | Stool consistency | Blood | Sum | DAI scores | Weight loss | Stool consistency | Blood | Sum | DAI scores | Weight loss | Stool consistency | Blood | Sum | DAI scores |
| 1 | 0 | 1 | 0 | 1 | 0.333333 | 0 | 1 | 0 | 1 | 0.333333 | 0 | 1 | 0 | 1 | 0.333333 | 0 | 1 | 0 | 1 | 0.333333 | 0 | 1 | 0 | 1 | 0.333333 | 0 | 1 | 0 | 1 | 0.333333 |
| 2 | 1 | 0 | 0 | 1 | 0.333333 | 1 | 1 | 0 | 2 | 0.666667 | 1 | 1 | 0 | 2 | 0.666667 | 1 | 2 | 0 | 3 | 1 | 1 | 1 | 0 | 2 | 0.666667 | 1 | 0 | 0 | 1 | 0.333333 |
| 3 | 0 | 2 | 1 | 3 | 1 | 0 | 2 | 1 | 3 | 1 | 0 | 2 | 1 | 3 | 1 | 0 | 2 | 0 | 2 | 0.666667 | 0 | 1 | 1 | 2 | 0.666667 | 0 | 2 | 1 | 3 | 1 |
| 4 | 0 | 3 | 1 | 4 | 1.333333 | 0 | 1 | 1 | 2 | 0.666667 | 0 | 3 | 0 | 3 | 1 | 0 | 3 | 1 | 4 | 1.333333 | 0 | 2 | 1 | 3 | 1 | 0 | 1 | 1 | 2 | 0.666667 |
| 5 | 0 | 2 | 0 | 2 | 0.666667 | 0 | 2 | 0 | 2 | 0.666667 | 0 | 2 | 0 | 2 | 0.666667 | 0 | 3 | 1 | 4 | 1.333333 | 0 | 3 | 1 | 4 | 1.333333 | 0 | 2 | 1 | 3 | 1 |
| 6 | 1 | 2 | 0 | 3 | 1 | 0 | 4 | 1 | 5 | 1.666667 | 0 | 4 | 1 | 5 | 1.666667 | 0 | 3 | 2 | 5 | 1.666667 | 0 | 4 | 0 | 4 | 1.333333 | 0 | 3 | 2 | 5 | 1.666667 |
| 7 | 1 | 3 | 1 | 5 | 1.666667 | 0 | 4 | 1 | 5 | 1.666667 | 0 | 3 | 2 | 5 | 1.666667 | 3 | 3 | 2 | 8 | 2.666667 | 0 | 4 | 3 | 7 | 2.333333 | 0 | 3 | 3 | 6 | 2 |
| 8 | 2 | 4 | 3 | 9 | 3 | 3 | 3 | 2 | 8 | 2.666667 | 2 | 3 | 3 | 8 | 2.666667 | 1 | 3 | 3 | 7 | 2.333333 | 1 | 3 | 2 | 6 | 2 | 1 | 3 | 2 | 6 | 2 |
| 9 | 3 | 4 | 3 | 10 | 3.333333 | 3 | 3 | 1 | 7 | 2.333333 | 3 | 4 | 2 | 9 | 3 | 2 | 3 | 2 | 7 | 2.333333 | 2 | 4 | 3 | 9 | 3 | 2 | 4 | 2 | 8 | 2.666667 |
| 10 | 3 | 4 | 2 | 9 | 3 | 3 | 3 | 2 | 8 | 2.666667 | 3 | 4 | 2 | 9 | 3 | 3 | 2 | 3 | 8 | 2.666667 | 3 | 3 | 2 | 8 | 2.666667 | 3 | 4 | 1 | 8 | 2.666667 |

Supplementary Table 4. The original data of each indicator of DAI scores in EBNH group

| Day | EBNH Group | | | | | | | | | | | | | | | | | | | | | | | | | | | | | |
| --- | --- | --- | --- | --- | --- | --- | --- | --- | --- | --- | --- | --- | --- | --- | --- | --- | --- | --- | --- | --- | --- | --- | --- | --- | --- | --- | --- | --- | --- | --- |
|  | 1 | | | | | 2 | | | | | 3 | | | | | 4 | | | | | 5 | | | | | 6 | | | | |
|  | Weight loss | Stool consistency | Blood | Sum | DAI scores | Weight loss | Stool consistency | Blood | Sum | DAI scores | Weight loss | Stool consistency | Blood | Sum | DAI scores | Weight loss | Stool consistency | Blood | Sum | DAI scores | Weight loss | Stool consistency | Blood | Sum | DAI scores | Weight loss | Stool consistency | Blood | Sum | DAI scores |
| 1 | 0 | 1 | 0 | 1 | 0.333333 | 0 | 1 | 0 | 1 | 0.333333 | 0 | 1 | 0 | 1 | 0.333333 | 0 | 1 | 0 | 1 | 0.333333 | 0 | 1 | 0 | 1 | 0.333333 | 0 | 1 | 0 | 1 | 0.333333 |
| 2 | 0 | 1 | 0 | 1 | 0.333333 | 0 | 2 | 0 | 2 | 0.666667 | 0 | 2 | 1 | 3 | 1 | 0 | 3 | 1 | 4 | 1.333333 | 0 | 1 | 1 | 2 | 0.666667 | 0 | 2 | 0 | 2 | 0.666667 |
| 3 | 1 | 2 | 0 | 3 | 1 | 0 | 3 | 0 | 3 | 1 | 0 | 1 | 1 | 2 | 0.666667 | 0 | 2 | 1 | 3 | 1 | 0 | 3 | 1 | 4 | 1.333333 | 0 | 1 | 1 | 2 | 0.666667 |
| 4 | 0 | 2 | 0 | 2 | 0.666667 | 0 | 1 | 1 | 2 | 0.666667 | 0 | 2 | 0 | 2 | 0.666667 | 0 | 3 | 1 | 4 | 1.333333 | 1 | 2 | 1 | 4 | 1.333333 | 0 | 2 | 0 | 2 | 0.666667 |
| 5 | 1 | 2 | 1 | 4 | 1.333333 | 0 | 1 | 1 | 2 | 0.666667 | 0 | 2 | 0 | 2 | 0.666667 | 0 | 2 | 0 | 2 | 0.666667 | 1 | 1 | 1 | 3 | 1 | 1 | 1 | 0 | 2 | 0.666667 |
| 6 | 1 | 1 | 1 | 3 | 1 | 0 | 2 | 1 | 3 | 1 | 0 | 3 | 1 | 4 | 1.333333 | 0 | 2 | 1 | 3 | 1 | 1 | 2 | 1 | 4 | 1.333333 | 0 | 4 | 2 | 6 | 2 |
| 7 | 2 | 2 | 1 | 5 | 1.666667 | 0 | 2 | 1 | 3 | 1 | 0 | 3 | 3 | 6 | 2 | 0 | 4 | 2 | 6 | 2 | 1 | 4 | 2 | 7 | 2.333333 | 1 | 3 | 3 | 7 | 2.333333 |
| 8 | 3 | 3 | 2 | 8 | 2.666667 | 1 | 3 | 3 | 7 | 2.333333 | 1 | 4 | 3 | 8 | 2.666667 | 2 | 4 | 3 | 9 | 3 | 1 | 4 | 2 | 7 | 2.333333 | 2 | 3 | 3 | 8 | 2.666667 |
| 9 | 3 | 3 | 1 | 7 | 2.333333 | 2 | 3 | 2 | 7 | 2.333333 | 2 | 4 | 3 | 9 | 3 | 3 | 4 | 4 | 11 | 3.666667 | 2 | 4 | 3 | 9 | 3 | 2 | 3 | 2 | 7 | 2.333333 |
| 10 | 2 | 3 | 2 | 7 | 2.333333 | 3 | 4 | 1 | 8 | 2.666667 | 3 | 3 | 2 | 8 | 2.666667 | 4 | 4 | 3 | 11 | 3.666667 | 3 | 4 | 3 | 10 | 3.333333 | 2 | 3 | 2 | 7 | 2.333333 |

Supplementary Table 5. The original data of each indicator of DAI scores in EBNM group

| Day | EBNM Group | | | | | | | | | | | | | | | | | | | | | | | | | | | | | |
| --- | --- | --- | --- | --- | --- | --- | --- | --- | --- | --- | --- | --- | --- | --- | --- | --- | --- | --- | --- | --- | --- | --- | --- | --- | --- | --- | --- | --- | --- | --- |
|  | 1 | | | | | 2 | | | | | 3 | | | | | 4 | | | | | 5 | | | | | 6 | | | | |
|  | Weight loss | Stool consistency | Blood | Sum | DAI scores | Weight loss | Stool consistency | Blood | Sum | DAI scores | Weight loss | Stool consistency | Blood | Sum | DAI scores | Weight loss | Stool consistency | Blood | Sum | DAI scores | Weight loss | Stool consistency | Blood | Sum | DAI scores | Weight loss | Stool consistency | Blood | Sum | DAI scores |
| 1 | 0 | 1 | 0 | 1 | 0.333333 | 0 | 1 | 0 | 1 | 0.333333 | 0 | 1 | 0 | 1 | 0.333333 | 0 | 1 | 0 | 1 | 0.333333 | 0 | 1 | 0 | 1 | 0.333333 | 0 | 1 | 0 | 1 | 0.333333 |
| 2 | 0 | 1 | 1 | 2 | 0.666667 | 0 | 1 | 0 | 1 | 0.333333 | 0 | 1 | 0 | 1 | 0.333333 | 0 | 2 | 0 | 2 | 0.666667 | 0 | 2 | 1 | 3 | 1 | 0 | 3 | 1 | 4 | 1.333333 |
| 3 | 0 | 2 | 0 | 2 | 0.666667 | 0 | 1 | 1 | 2 | 0.666667 | 0 | 3 | 1 | 4 | 1.333333 | 0 | 2 | 1 | 3 | 1 | 0 | 2 | 1 | 3 | 1 | 0 | 1 | 1 | 2 | 0.666667 |
| 4 | 0 | 3 | 1 | 4 | 1.333333 | 0 | 2 | 1 | 3 | 1 | 0 | 2 | 1 | 3 | 1 | 0 | 3 | 1 | 4 | 1.333333 | 0 | 1 | 1 | 2 | 0.666667 | 0 | 2 | 1 | 3 | 1 |
| 5 | 0 | 3 | 2 | 5 | 1.666667 | 0 | 3 | 1 | 4 | 1.333333 | 0 | 4 | 1 | 5 | 1.666667 | 0 | 4 | 0 | 4 | 1.333333 | 0 | 3 | 0 | 3 | 1 | 1 | 3 | 1 | 5 | 1.333333 |
| 6 | 0 | 4 | 1 | 5 | 1.666667 | 0 | 4 | 3 | 7 | 2.333333 | 0 | 4 | 2 | 6 | 2 | 0 | 2 | 1 | 3 | 1 | 0 | 3 | 2 | 5 | 1.666667 | 0 | 2 | 0 | 2 | 0.666667 |
| 7 | 1 | 4 | 2 | 7 | 2.333333 | 1 | 4 | 2 | 7 | 2.333333 | 1 | 3 | 2 | 6 | 2 | 1 | 4 | 2 | 7 | 2.333333 | 1 | 4 | 2 | 7 | 2.333333 | 1 | 4 | 3 | 8 | 2.666667 |
| 8 | 2 | 4 | 3 | 9 | 3 | 1 | 3 | 3 | 7 | 2.333333 | 2 | 3 | 2 | 7 | 2.333333 | 2 | 3 | 2 | 7 | 2.333333 | 1 | 3 | 3 | 7 | 2.333333 | 2 | 4 | 2 | 8 | 2.666667 |
| 9 | 3 | 2 | 1 | 6 | 2 | 3 | 4 | 3 | 10 | 3.333333 | 3 | 3 | 1 | 7 | 2.333333 | 3 | 4 | 2 | 9 | 3 | 2 | 4 | 1 | 7 | 2.333333 | 3 | 4 | 2 | 9 | 3 |
| 10 | 3 | 4 | 2 | 9 | 3 | 4 | 4 | 2 | 10 | 3.333333 | 3 | 4 | 2 | 9 | 3 | 3 | 3 | 3 | 9 | 3 | 3 | 4 | 2 | 9 | 3 | 3 | 3 | 2 | 8 | 2.666667 |

Supplementary Table 6. The original data of each indicator of DAI scores in EBNL group

| Day | EBNL Group | | | | | | | | | | | | | | | | | | | | | | | | | | | | | |
| --- | --- | --- | --- | --- | --- | --- | --- | --- | --- | --- | --- | --- | --- | --- | --- | --- | --- | --- | --- | --- | --- | --- | --- | --- | --- | --- | --- | --- | --- | --- |
|  | 1 | | | | | 2 | | | | | 3 | | | | | 4 | | | | | 5 | | | | | 6 | | | | |
|  | Weight loss | Stool consistency | Blood | Sum | DAI scores | Weight loss | Stool consistency | Blood | Sum | DAI scores | Weight loss | Stool consistency | Blood | Sum | DAI scores | Weight loss | Stool consistency | Blood | Sum | DAI scores | Weight loss | Stool consistency | Blood | Sum | DAI scores | Weight loss | Stool consistency | Blood | Sum | DAI scores |
| 1 | 0 | 1 | 0 | 1 | 0.333333 | 0 | 1 | 0 | 1 | 0.333333 | 0 | 1 | 0 | 1 | 0.333333 | 0 | 1 | 0 | 1 | 0.333333 | 0 | 1 | 0 | 1 | 0.333333 | 0 | 1 | 0 | 1 | 0.333333 |
| 2 | 0 | 2 | 0 | 2 | 0.666667 | 0 | 1 | 1 | 2 | 0.666667 | 0 | 2 | 0 | 2 | 0.666667 | 0 | 2 | 1 | 3 | 1 | 0 | 3 | 0 | 3 | 1 | 0 | 3 | 1 | 4 | 1.333333 |
| 3 | 1 | 1 | 1 | 3 | 1 | 0 | 2 | 1 | 3 | 1 | 0 | 1 | 1 | 2 | 0.666667 | 0 | 3 | 0 | 3 | 1 | 0 | 1 | 1 | 2 | 0.666667 | 0 | 2 | 1 | 3 | 1 |
| 4 | 1 | 1 | 1 | 3 | 1 | 0 | 3 | 0 | 3 | 1 | 0 | 2 | 0 | 2 | 0.666667 | 0 | 2 | 1 | 3 | 1 | 0 | 3 | 1 | 4 | 1.333333 | 0 | 1 | 1 | 2 | 0.666667 |
| 5 | 1 | 2 | 1 | 4 | 1.333333 | 0 | 3 | 1 | 4 | 1.333333 | 0 | 3 | 1 | 4 | 1.333333 | 0 | 2 | 1 | 3 | 1 | 0 | 2 | 1 | 3 | 1 | 0 | 2 | 1 | 3 | 1 |
| 6 | 1 | 3 | 1 | 5 | 1.666667 | 0 | 3 | 2 | 5 | 1.666667 | 0 | 3 | 2 | 5 | 1.666667 | 1 | 3 | 2 | 6 | 2 | 1 | 2 | 1 | 4 | 1.333333 | 0 | 3 | 1 | 4 | 1.333333 |
| 7 | 1 | 4 | 4 | 9 | 3 | 1 | 4 | 3 | 8 | 2.666667 | 0 | 4 | 2 | 6 | 2 | 1 | 4 | 3 | 8 | 2.666667 | 0 | 4 | 2 | 6 | 2 | 0 | 4 | 3 | 7 | 2.333333 |
| 8 | 1 | 4 | 3 | 8 | 2.666667 | 2 | 4 | 4 | 10 | 3.333333 | 1 | 4 | 3 | 8 | 2.666667 | 2 | 4 | 3 | 9 | 3 | 2 | 4 | 4 | 10 | 3.333333 | 0 | 4 | 4 | 8 | 2.666667 |
| 9 | 3 | 4 | 3 | 10 | 3.333333 | 3 | 4 | 3 | 10 | 3.333333 | 2 | 4 | 4 | 10 | 3.333333 | 3 | 4 | 4 | 11 | 3.666667 | 3 | 4 | 4 | 11 | 3.666667 | 2 | 4 | 3 | 9 | 3 |
| 10 | 4 | 4 | 4 | 12 | 4 | 3 | 3 | 3 | 9 | 3 | 3 | 4 | 3 | 10 | 3.333333 | 4 | 4 | 3 | 11 | 3.666667 | 3 | 4 | 2 | 9 | 3 | 3 | 4 | 3 | 10 | 3.333333 |
